# Consumers’ Expectations about Meat from Surgical Castrated or Immunocastrated Male and Female Iberian Pigs

**DOI:** 10.3390/ani12040468

**Published:** 2022-02-14

**Authors:** Maria Font-i-Furnols, Anna Claret, Luis Guerrero, Antoni Dalmau

**Affiliations:** 1 IRTA Food Quality and Technology, Finca Camps i Armet s/n, 17121 Monells, Spain; lluis.guerrero@irta.cat; 2 IRTA Animal Welfare, Veïnat de Sies s/n, 17121 Monells, Spain; antoni.dalmau@irta.cat

**Keywords:** disconfirmation, assimilation, attitudes, beliefs, cognitive dissonance

## Abstract

**Simple Summary:**

Pig castration is a common practice in the Iberian breed (boar and gilts), known for the quality of its products. This work studies the effect of pig castration (surgically castrated females and males, immunocastrated females and males and entire females) on the sensory expectations and acceptability of the meat. Attitudes and beliefs towards castration and immunocastration were also evaluated. The study was carried out in Madrid and Barcelona, where consumers (*n* = 252) evaluated meat from the five sex types in blind and informed conditions. Results showed that consumers could be classified into three groups as “Indifferent”, “Against castration and immunocastration” and “Against immunocastration”. Meat from castrated males had higher overall liking scores than the other types of meat. Information provided to the consumers, together with their expectations, affects the overall liking scores. Thus, this needs to be considered to determine the best marketing strategy according to the type of pork produced.

**Abstract:**

A common practice in Iberian pigs is the castration of both males and females, and it can be carried out surgically or by immunization against gonadotropin-releasing factor (GnRF). The aim of this work was to determine consumers’ overall liking and expectations towards Iberian pork from five different sex types (castrated females, entire females, GnRF-vaccinated females, castrated males and GnRF-vaccinated males), as well as to know the attitudes and beliefs of consumers towards castration and immunocastration. Loins from 83 Iberian pigs were collected and evaluated by 252 consumers in Barcelona and Madrid. Consumers evaluated the five types of meat in three situations: blind condition (tasting the product), expectations (without tasting) and informed condition. Finally, attitudes and beliefs towards castration and immunocastration were also determined. Results distinguished three segments of consumers labeled as “Indifferent”, “Against castration and immunocastration” and “Against immunocastration”. Meat from castrated males had higher overall liking scores in the blind condition. Expectations towards pork depending on its sex type affect consumer-informed acceptability; thus, it is important to consider marketing strategies to avoid or direct the effect of the information provided on the acceptability of the pork.

## 1. Introduction

The Iberian pig is an autochthonous breed from the Iberian Peninsula characterized by its high-quality meat and meat products [1] and the high amount of fat [2,3,4]. The Iberian pig is perfectly adapted to its natural environment; a wooded pasture-land called La Dehesa, which is characterized by oak forest (*Quercus rotundifolia* and *Quercus suber*), grasses and legumes, among other herbaceous species [5]. In the traditional production system, Iberian piglets are weaned at two months old, and then they are usually mixed in large pastures. In these areas, they are fed a combination of concentrate and natural resources until approximately 90–115 kg of body weight [2]. The late fattening phase is carried out in free-range in La Dehesa and is called “montanera”. It takes place in the late autumn and winter when pigs are able to eat acorns from the oaks and cork oaks, as well as other natural products from La Dehesa pastures [6]. During “montanera”, pigs gain approximately 50–60 kg [5,6,7]. Thus, pigs that expend the late fatting phase freely in “montanera” are recognized with a distinctive mask of best quality for their products [8]. The age of the animals at the end of the growing period can vary from 10 to 24 months old [2], and they are fattened until they reach 160–180 kg to achieve the minimum carcass weight of 115 kg required by law RD 1469/2007 [9]. Currently, the term “Iberian” meat can be used when it comes from a pig with a minimum of 50% Iberian genetics, while the mother must be 100% Iberian [9].

It has been reported that the beginning of puberty of an Iberian female is at an average of 6 to 7 months [5,10], and in males, it is around 7 to 9 months of age [11]. According to this, male and female pigs are reared free-range for several months after sexual maturity. Consequently, male castration is required to avoid boar taint in their final product [12], because it would negatively affect the quality. Meanwhile, females are spayed, mainly to avoid pregnancies due to the presence of wild boars in La Dehesa [13], which can affect the production cycle and cause sanitary problems. However, spaying is regulated by Spanish national law RD 1221/2009 [14], and the castration of males would be limited in the EU. Consequently, alternatives to this surgery, such as vaccination against gonadotropin-releasing factor (GnRF) (immunocastration), are being studied. Immunocastration has long been used in intensively-bred white pigs [15] and may be a good option for free-ranging Iberian pigs [12], as it can suppress oestrus and sexual behavior [13,16].

Several types of consumers can be found when attitudes towards animal welfare are considered, characterized by different focuses on the importance of this issue. Furthermore, consumer attitudes towards immunocastration are highly variable and are influenced by the way the information is provided to the consumers and the type of information available [17,18]. Immunocastration was a good option by consumers from different countries compared to other alternatives towards surgical castration without anesthesia [17,19,20,21]. A recent study carried out in 16 countries [22] showed that, after providing information, immunocastration of entire males was accepted by 71% of consumers. However, there is a lack of information about consumer attitudes, beliefs and preferences when tasting actual samples from immunocastrated or surgical castrated females. In general, the main concern of consumers about immunocastration is food safety issues, mainly related to the use of hormones and the residues from medication [23]. Nevertheless, the taste and odor of the meat were the most important characteristics that consumers considered when buying and consuming pork [24]. In this line, meat from immunocastrated and surgically castrated males and meat from gilts, evaluated in blind conditions by consumers, was more accepted than that of entire males, even if entire males have low levels of androstenone [25]. This can be explained because meat from entire males was less sweet and juicy and harder with higher androstenone and skatole odor and flavor [26]. On the other hand, Martínez-Mancipe et al. [12] reported that meat from immunocastrated males had higher rancidity (odor and flavor) scores than those from immunocastrated females, entire females or surgically castrated males and females. The same authors also reported that meat from immunocastrated male pigs was also harder than that from immunocastrated females and less sweet than that from entire females.

Expectations are key drivers for the acceptance or rejection of a product and depend on consumers’ previous experiences, the information available at the moment of purchase and the context of consumption [27,28]. During consumption, the sensory characteristics of the product play an important role. However, expectations may also affect product perception [28]. After tasting the product, expectations can or cannot be satisfied. When expectations are not met, the individual response can be explained by several theories, with the assimilation or cognitive dissonance theory being the most common. According to this theory, consumers try to diminish disconfirmation by changing the perception of the product to minimize the differences between the expected and real perceptions [29].

The aim of this paper was to find out and compare consumers’ overall liking and expectations towards Iberian pork from five different sex types (castrated females (CF), entire females (EF), GnRF-vaccinated females (IF), castrated males (CM) and GnRF-vaccinated males (IM)), as well as to know the attitudes and beliefs of consumers towards castration and immunocastration.

## 2. Materials and Methods

### 2.1. Participants

Consumer participation was structured into two parts, (a) qualitative focus groups and (b) quantitative questionnaire and sensory evaluation.

(a) In the qualitative group, four focus group discussions of eight regular meat consumers each (*n* = 32) were carried out, two in Barcelona and two in Madrid, in order to collect their beliefs about castration and animal welfare. All participants were involved in food purchasing and preparation at home and were recruited considering age (between 20 and 70 years), gender (50% men and 50% women) and meat consumption (minimum of twice a week).
(b) For the quantitative part, a sample of 252 regular meat consumers was recruited in Barcelona (*n* = 151) and Madrid (*n* = 101). A probabilistic sampling of the participants, by quotas, was carried out by phone, using a filter questionnaire specifically designed for this study, using the information obtained in the focus group. Quotas included age (between 20 and 70 years) and gender (minimum 25% men). Only those consumers involved in food purchase and preparation in their household and who stated that they eat meat at least twice a week were selected.

### 2.2. Samples

For the sensory evaluation, a total of 83 loins were used, 19 from surgically castrated females (CF), 8 from entire females (EF), 16 from immunocastrated females (IF), 19 from surgically castrated males (CM) and 21 from immunocastrated males (IM). Castration of male pigs was carried out at the first week of age and castration of females at the 6th week of age. The vaccination against GnRF (immunocastration) was carried out by the subcutaneous application of three doses of 2 mL of IMPROVAC^®^ (Zoetis, Madrid, Spain) at 11, 12 and 14 months old, in both male (IM) and female (IF) pigs.

Loins were from Iberian pigs of Valdesequera strain reared in extensive conditions, finished in oak forest of montanera (Valdesequera, Extremadura, Spain) and slaughtered in a commercial abattoir, previously electrically stunned, at the age of 15–16 months and live weight of 155 ± 8.4 kg [12]. Carcasses were kept at 3 °C until the next day, where, at the cutting room, they were placed in the slaughterhouse building, left loins were mechanically sliced (Meat Slicer 350 VK BV Dual, Marconi, Italy) at 0.5 cm thick and kept in sealed bags (12 μm metallic polyester/110 μm polyethylene multilayer; oxygen permeability: b1.5 cm^3^/m^2^, 158/24 h; water-vapor permeability: b1 g/m^2^, 159/24 h; Sacoliva, S.L., Castellar del Vallès, Spain) at −20 ± 2 °C for a maximum period of 1 month before being used for the consumer evaluation.

### 2.3. Expectations and Overall Liking

Twenty-four hours before the sensory evaluation, samples were thawed at 4 ± 2 °C in a refrigerated room. Five slices of *Longissimus thoracis* were obtained and divided into 2 pieces of similar size. Pieces were individually wrapped with aluminum sheets, coded with a 3-digit random number, and cooked in a preheated convection HBA 74 A 168 250E oven (Bosch, Barcelona, Spain) at 120 ± 2 °C for 10 min. The five meat samples (CF, EF, IF, CM and IM) were presented to participants in a sequential monadic way, one every 5 min, balancing the first-order and the carry-over effects as much as possible, according to Macfie et al. [30]. The evaluation of the samples was performed in a sensory testing room with ten individual booths at 20 ± 2 °C under F8W/D daylight conditions (Sylvania, United Kingdom). Participants were assisted by two researchers who provided instructions on how to proceed. Mineral water and unsalted crisp bread were provided to participants to rinse their mouths between samples.

The evaluation of the samples was carried out in three different conditions of information [28,31]:

- Blind-condition-liking: consumers received the five samples codified with a three-digit code and were asked to rate their overall liking by means of a semi-structured 10 cm lineal scale anchored in the two extremes (0 = I extremely dislike and 10 = I extremely like).
- Expectations: raised only by the information regarding the sex type of the animals: consumers were only provided with the name of each sex type studied. Next, they were asked to rate their expected liking for the five meat samples by means of a semi-structured 10 cm lineal scale anchored in the two extremes (0 = I think I would dislike it extremely and 10 = I think I would like it extremely.
- Effect of information on perceived liking: consumers received the five samples identified with the name of the treatment (sex type) and were asked to rate their overall liking by means of a semi-structured 10 cm lineal scale anchored in the two extremes (0 = I extremely dislike and 10 = I extremely like). For each sample, they were also asked to evaluate their willingness to buy in a semi-structured 10 cm lineal scale anchored in the two extremes (0 = Sure that I will not buy it and 10 = Sure I will buy it).

After tasting the samples, consumers completed a questionnaire concerning beliefs and attitudes towards animal welfare related to castration.

### 2.4. Focus Groups and Questionnaire

The design of the questionnaire was based on the information obtained by means of focus groups (qualitative approach) performed to identify key beliefs about castration and animal welfare. Focus group sessions lasted 1.5–2 h and were structured in three main stages: generic discussion on meat consumption (frequency, habits, place of purchase), motives/advantages and barriers/disadvantages of meat consumption and castration (overall knowledge, attitudes, beliefs and effect on meat quality). Some pictures were presented to participants during the sessions to enrich the discussion. The same experienced moderator conducted the focus group sessions in both geographic locations. An observer was also present to take notes. Each session was audio and video recorded for deeper qualitative analysis.

The final questionnaire items were derived from the information obtained from 14 questions related to castration (10 and 4 items assessing beliefs and attitudes, respectively) measured by means of a 7-point Likert scale (1 = strongly disagree; 4 = neither agree nor disagree; 7 = strongly agree). Questions and possible answers are presented in results section. Socio-demographic data (gender, age, education and perceived economic situation, see Table 1) were also recorded.

### 2.5. Statistical Analysis

Differences in expectations and overall liking were evaluated by means of the MIXED procedure of the SAS software (v.9.4., SAS Institute Inc., Cary, NY, USA). The model included the sex of the animal according to the castration technique used (CF, EF, IF, CM and IM), the type of test (blind, expectations, informed) and its interaction as fixed effects. The tasting session was included as a blocking variable. Repeated measurements were considered since the same consumer evaluated the different sex types in three different conditions. The analysis was performed for the pooled sample of consumers first and then for each of the identified clusters.

Clusters were determined with the XLStat (version 2021.2.2., Addinsoft, NY, USA) software using Euclidian distance and the Ward method and considering the scores obtained for the five different types of meat according to sex type in the three conditions of information. The number of clusters to retain was determined based on the obtained dendrogram, considering the homogeneity within and among the segments and the principle of parsimony [32]. A discriminant analysis was performed to validate the number of clusters retained by checking how many individuals were properly classified in their corresponding cluster (confusion matrix).

An analysis of variance was carried out for the purchasing intention, considering the sex type as a fixed effect for the pooled sample and by cluster. Furthermore, an analysis of variance was performed for the attitudes and beliefs, considering clusters as fixed effects.

A Tukey test was applied to find differences between least squared means. Significance was fixed at *p* < 0.05.

## 3. Results

### 3.1. Consumers Demographic Characteristics

Consumers were segmented into three different Clusters according to the scores given to the samples in the three conditions of information studied. Cluster 1 and 2 had the same size (*n* = 79) and Cluster 3 was slightly numerous (*n* = 94) (Table 1).

Overall, participants were balanced by gender (46% male vs. 54% female). Males were slightly underestimated in Barcelona and in Clusters 2 and 3 but accomplished the required quotas (minimum 25% men). Regarding age, globally and within city, consumers followed the Spanish distribution.

Overall, 36.7% of the consumers considered having an accommodated economic situation, 30.7% a difficult economic situation and 32.6% neither accommodated nor difficult. In Barcelona, a difficult economic situation was declared by 28.6% of the consumers and in Madrid, by 33.6%. By clusters, this percentage varied between 29.8 (Cluster 3) and 30.4% (Cluster 1). Consumers that considered having an accommodated economic situation were 40.7% in Barcelona and 30.7% in Madrid. This percentage was 26.9% in Cluster 2 and 43.6% in Cluster 3, being in between in Cluster 2 (38.0%).

### 3.2. Consumers Loins’ Acceptability by Sex Type for Each Condition of Information

**Figure 1:**
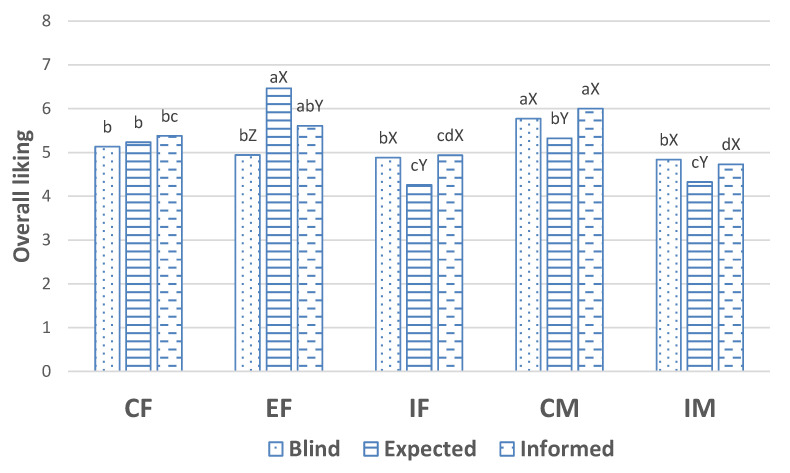
Acceptability of pork from surgically castrated females (CF), entire females (EF), immunocastrated females (IF), surgically castrated males (CM) and immunocastrated males (IM) obtained from the evaluation meat in blind condition, as expectations and from the evaluation of meat in informed conditions according to a scale from 0 (Extremely dislike/I think I would dislike it extremely) to 10 (Extremely like/I think I would like it extremely). Different letters, a,b,c,d indicate significant differences between sex type within condition of information, and different letters X,Y,Z indicate significant differences between conditions of information and within sex type.

The overall liking in the blind condition was significantly (*p* < 0.05) higher for meat samples from CM than for those from the other sex types (Figure 1). However, when consumers tasted the meat in informed condition, overall liking was higher (*p* < 0.05) in loins from CM than those from CF, IF and IM and not significantly different from those of EF. The lowest (*p* < 0.05) overall acceptability was for loins from IF and IM. The expected overall acceptability was highest (*p* < 0.05) for loins from EF, intermediate (*p* < 0.05) for loins from CM and CF and lower in loins from IF and IM (*p* < 0.05). Thus, when all the consumers were considered together, expectations for meat from entire pigs was the highest, followed by meat from surgically castrated pigs and finally, the lowest expectancies were for meat from immunocastrated pigs, both males and females. Thus, in an overall sense, participants in our study should be regarded as “against immunocastration and slightly against surgical castration”.

Three different clusters of consumers were obtained when their overall acceptability (in blind and informed conditions) and expectations were considered.

**Figure 2:**
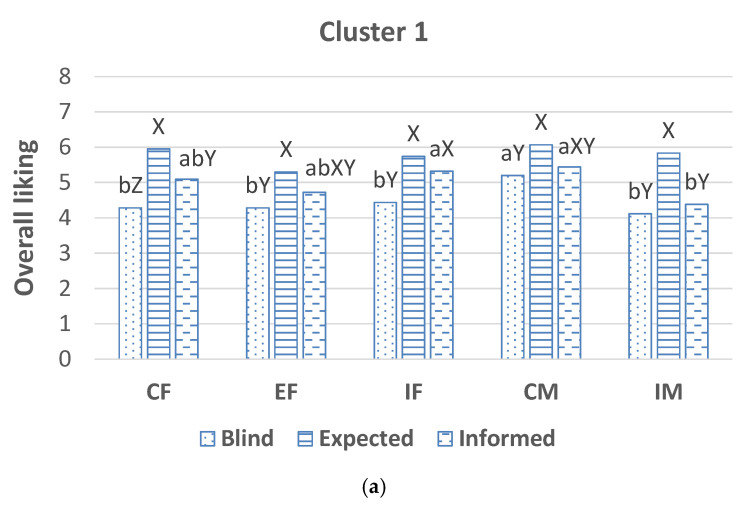

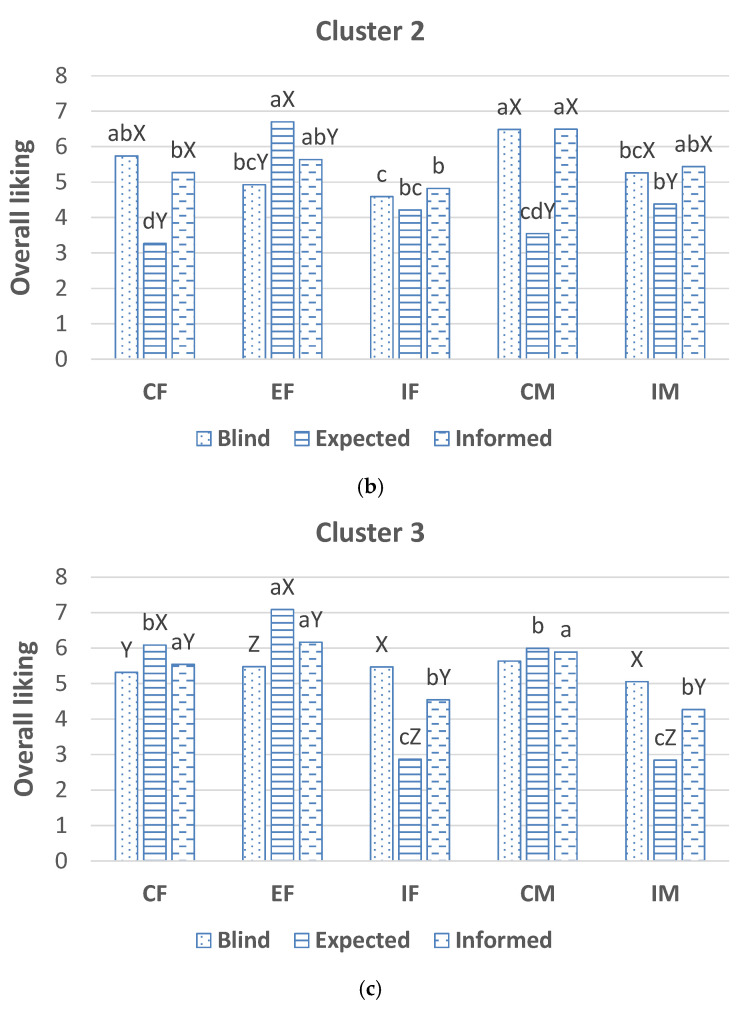
Acceptability scores by consumers of Cluster 1 (**a**), Cluster 2 (**b**) and Cluster 3 (**c**) of pork from surgically castrated females (CF), entire females (EF), immunocastrated females (IF), surgically castrated males (CM) and immunocastrated males (IM) obtained from the evaluation in blind conditions, as expectations and from the evaluation of meat in informed conditions according to a scale from 0 (Extremely dislike/I think I would dislike it extremely) to 10 (Extremely like/I think I would like it extremely). Different letters, a,b,c,d indicate significant differences between sex type within condition of information, and different letters X,Y,Z indicate significant differences between conditions of information and within sex type.

Consumers from Cluster 1 (Figure 2a) scored overall acceptability of CM loins higher than the others when evaluated in blind conditions. When they evaluated the overall liking in informed conditions, this was higher for loins from CM and IF than those from IM. Overall liking of EF and CF loins were intermediate and not significantly different for the others. Their expectations were not significantly affected by the different sex types. Thus, this segment of consumers was labeled as “Indifferent towards castration and immunocastration” or with no preferences.

Regarding consumers from Cluster 2 (Figure 2b), in blind conditions, they scored overall liking higher (*p* < 0.05) in loins from CM than those from EF, IF and IM. Loins from CF were in between and significantly (*p* < 0.05) higher than those from IF. When scoring the overall acceptability after tasting loins with information about its sex type, loins from CM were scored higher (*p* < 0.05) than those from CF and IF, with those from EF and IM being in between. EF loins were in the area of “I think I would like” while the others were in the area of “I think I neither would like nor dislike”—“I think I would dislike”. This cluster was named “Against castration and immunocastration”.

Finally, consumers from Cluster 3 (Figure 2c) did not find significant (*p* > 0.05) differences in overall liking when evaluated in blind conditions. However, in informed conditions, loins from CF, EF and CM had significantly (*p* < 0.05) higher overall liking scores than those from IF and IM. In fact, the expected likings of loins from EF were higher than those from CF and CM, but all of them were in the region of “I think I would like”, while the expected liking of loins from IF and EM were significantly (*p* < 0.05) lower than the others and in the region of “I think I would dislike”. Thus, consumers from this cluster were classified as “Against immunocastration”.

### 3.3. Consumers’ Purchasing Intentions when Informed

The consumers’ purchasing intention of the loins after tasting the samples knowing their sex type, is presented in Table 2. When consumers were considered as a pool, purchasing intention of loins from EF and CM were higher than those from IF and IM. For the “Indifferent” consumers (Cluster 1), purchasing intentions were higher for loins from CM than from those from IM, being in between for CF, EF and IF. Consumers “Against castration and immunocastration” (Cluster 2) stated a higher purchase intention for loins from CM and EF than those from IF, with loins from CF and IM being in between. Finally, consumers “Against immunocastration” (Cluster 3) showed a lower purchasing intention for loins from immunocastrated animals, i.e., IF and IM, than those from the other sex types.

### 3.4. Consumers’ Attitudes and Beliefs towards Castration

Although all the mean values were close to the intermediate point of the scale (4), in an overall sense, consumers believe that castration causes pain to the animal (4.5) and that chemical castration worsens meat quality (4.7). However, they are willing to pay a little more for meat from castrated animals (4.5). Regarding the remaining beliefs studied, the consumer answer was neutral (between 3.8 and 4.4). Concerning attitudes, globally, consumers think that castrating animals is artificial (4.9) and that castration of pigs is easy (3.5). Consumers provided a neutral response (4.0–4.1) to the rest of the statements studied.

Attitudes and beliefs towards castration are presented by cluster in Table 3. Consumers from Cluster 2 and 3 disagreed significantly (*p* > 0.05) more than those from Cluster 1 in the statements “Meat of castrated animals is of higher quality”, “I am willing to pay a little more for meat from castrated animals”, “I prefer to eat meat of castrated animals” and “Castration with vaccines improves meat quality”. Consumers “Against immunocastration” (Cluster 3) agreed more (*p* < 0.05) than those from Cluster 1 in the statement “Chemical castration worsens meat quality”. Consumers from all the clusters agreed in the fact that “Castration causes pain to the animal”, “Meat of castrated animals is more expensive”, “Castration is necessary”, “Meat of castrated animals is leaner” and “Castration is savage”.

Regarding attitudes, consumers from Cluster 2 and 3 considered castration less beneficial and consequently perceived castration worse than those from Cluster 1. Moreover, consumers from Cluster 2 considered castration as more difficult than those from Clusters 1 and 3. No differences between clusters were observed regarding the natural or artificial perception of castration.

## 4. Discussion

### 4.1. Sensory Acceptability of Meat from Different Sex Types

Castration of male pigs is a common practice to improve management and eliminate an important sensory problem, boar taint, which is associated with entire males and can affect the consumers’ acceptability [26,33]. Male pigs’ surgical castration also has an effect on the quality of the fat and meat [34], and this might impact its sensory acceptability. Gilts’ castration is a less common practice, only carried out in some type of gilts, like those from Iberian [3]. Because of that, the effect of castration of gilts on the pork quality and its acceptability by consumers is less known. However, some works show non-significant differences in the quality of pork from females, immunocastrated and surgically castrated females [12,35,36].

The sensory evaluation of meat in blind conditions allows us to know the overall liking of the meat without the influence of the information provided. In general, meat from CM pigs had the highest overall liking scores, while non-significant differences were observed among pork from the other sex types evaluated. This difference is difficult to explain since meat from CM did not differ from meat from the other sex types in any of the odor (overall intensity, animal and skatole), flavor (metallic, sweet, animal and skatole) or texture (hardness, fibrousness, juiciness and crumbliness) attributes evaluated by a trained panel [12]. According to the trained panel, meat from IM had the highest rancid odor and flavor scores, and meat from CF had the highest overall flavor intensity scores, but the difference in these attributes detected by the panel did not seem to be high enough to create different acceptability scores by consumers [12]. In a previous study, Font i Furnols et al. did not report significant differences in the overall acceptability of meat from surgically castrated males, entire females and immunocastrated males. Furthermore, no differences in overall acceptability were reported by Aluwé et al. when consumers evaluated meat from surgically castrated and immunocastrated male pigs. In both studies, pigs were from white genetic lines, while in the present study, pigs were Iberian genotype. Maybe the high amount of intramuscular fat in the pigs of the present work, which was between 7.7 and 9.5% as reported by Martinez-Mancipe et al. [12], might have influenced the perception of the consumers, although it cannot explain differences in acceptability because there were no significant differences in the intramuscular fat content and marbling between sex types. Differences in the fatty acid content of pigs from different sex types have been reported [34,37,38], and it can affect the sensory quality of the meat and, consequently, consumers’ acceptability. However, this quality characteristic has not been evaluated in the present study.

Sensory acceptability scores are influenced by the beliefs and attitudes of consumers with respect to the information provided and previous experiences [27]. Thus, the knowledge of the sex type (informed condition) when tasting pork influences the sensory perception of the meat. This can be seen by the differences in scores given to the different meat types when evaluated in blind or informed conditions in the present work. Beliefs and attitudes affected mainly the scores of pork from IM and IF, which received lower scores than those from EF and CF, while in blind conditions, they were similar.

### 4.2. Expectations and Willingness to Purchase Meat from Different Sex Types

Consumers have expectations and opinions towards meat liking and the effect of the castration, either surgical or immunological, on the taste of pork, which are affected by the type of information provided [18,24]. In our work, no information was provided to the participants regarding pork production practices, advantages and disadvantages of production of entire, surgically castrated and immunocastrated pigs, animal welfare, meat quality or other issues. Thus, expectations towards the products assessed were due to previous knowledge and experiences of consumers, beliefs, attitudes and intentions as well as the information provided at the moment of the evaluation about the different types of meat (i.e., sex type), and were related to consumer satisfaction [29]. We opted for this approach to have a greater ecological validity of the results obtained since, in a real context of purchase and/or consumption, most consumers will not have a detailed description of the productive and economic implications of the different samples to be bought.

In the present work, three segments of consumers were identified, those “Indifferent towards castration and immunocastration”, those “Against castration and immunocastration” and those “Against immunocastration”. The clusters were obtained considering both the sensory scores of the meat in blind and informed conditions as well as the expectations. Considering the attitudes and beliefs towards castration, Tomasevic et al. identified a different cluster, composed of 22% eastern consumers, that was named “pro-castration”. Curiously, this cluster of consumers in favor of the castration has not been identified in the present study, although, when consumers tasted the pork loins, those from castrated animals obtained, in general, the highest scores.

This works shows that an important group of consumers (Cluster 1) were indifferent towards the effect of pork sex type on its overall liking and thought they would like all the loins at the same level. Moreover, their scores were in the intermediate part of the scale, which is the region equivalent to “I think I would neither like nor dislike” the meat, probably denoting insufficient comprehension or lack of knowledge of the effect of sex type on the liking of the meat. These consumers “Indifferent towards castration and immunocastration” did not meet their expectations for any type of pork evaluated because all the expected scores were higher than those obtained in blind conditions. Thus, they had a negative disconfirmation, which can cause product rejection. According to the assimilation theory of Issanchou [28], consumers that had disconfirmation try to reduce the discomfort that the disconfirmation produces by modifying their liking when information is provided. This is known as cognitive dissonance [39]. This phenomenon can be seen in the scores provided in informed conditions for CF and IF loins, which increased with respect to those obtained in blind conditions, being closer to the expected scores. Although the expectations were the same for all types of meat, after tasting them in informed conditions, their willingness to buy was higher for meat from CM, probably because the sensory scores were also higher for this type of meat, although not significantly different from those of CF, EF and IF.

When consumers were “Against castration and immunocastration” (Cluster 2), the highest expectations were for loins from EF. However, the expectations for EF meat were not met when they tasted the meat in blind conditions (negative disconfirmation), while the expectations for CF, CM and IM were achieved and overcome (positive disconfirmation). These consumers were not affected by the information about the type of pork since the scores obtained in informed conditions were not significantly different from those obtained in blind conditions. Thus, in this group of consumers, there was not an assimilation effect. According to these results, consumers classified as “Against castration and immunocastration” were not affected by the information provided. However, there would be the possibility that, if they had more experiences in informed conditions, they might be aware of this disconfirmation. This could lead them to revise and maybe change their expectation to adjust it to the “real” properties of the meat [40].

Consumers “Against immunocastration” (Cluster 3) had the lowest expectation scores for pork from IF and IM pigs. Nevertheless, for these types of meat, expectations were exceeded when tasting in blind conditions; thus, a positive disconfirmation occurred. In both cases, when consumers evaluated the meat in informed conditions, they tried to minimize the discomfort produced by this disconfirmation by reducing the scores of overall acceptability for IF and IM (assimilation effect). Probably because of this, willingness to purchase was lower for meat from IM and IF. Purchasing decisions must be based on the expectations toward the product [40] but also on the sensory experience. In the case of consumers “Against immunocastration”, expectations probably had a higher role in the purchasing decision than the sensory experience, since consumers seemed not to trust the pork sensory evaluation too much of meat from IM and IF pigs already immunocastrated. Thus, for these consumers, the provided information about the sex of the pig would be prejudicial in terms of the consumption of meat from immunocastrated boars and gilts. Providing information to consumers with respect to immunocastration would probably help improve the ideas they already have, and help improve understanding of this practice, as has been previously shown in other studies [19,22]. Expectations were also not filled for meat from CF and EF, but in this case, they were higher, and they were not achieved when tasting the meat (negative disconfirmation). For meat from EF, which had the highest expectations of overall liking, consumers tried to minimize this disconfirmation, thus, increasing their sensory score in the informed condition (assimilation effect).

### 4.3. Relation between Expectations and Attitudes and Beliefs towards Castration and Immunocastration

Consumers’ perception of a product is influenced by their attitudes and beliefs [41]. Some of the beliefs and attitudes that might affect the expectations have been analyzed (Table 3).

For some of the statements, differences were observed between clusters. Even though clusters were not obtained considering the responses to the beliefs and attitudes questions, they should have an important impact on consumer expectations towards meat from castrated and immunocastrated pigs. Thus, consumers “Indifferent towards castration and immunocastration” had the highest agreement with the statements “Meat of castrated pigs are of higher quality”, “I am willing to pay a little more for meat from castrated animals”, “I prefer to eat meat from castrated animals” and “Castration with vaccines improves meat quality”. These beliefs explained the lower expectations of consumers from Clusters 2 and 3 towards meat from castrated and/or immunocastrated pigs. Furthermore, the statement “Chemical castration worsens meat quality” had the highest scores by consumers from Cluster 3 (“Against immunocastration”) and the lowest for consumers from Cluster 1 (“Indifferent”). Because of the lack of information provided and the expected low knowledge of consumers towards castration and immunocastration [17,24,42], there was probably confusion between chemical castration and castration with vaccines that could not be differentiated by most consumers. The same pattern can be observed with the attitudes evaluated. Consumers “Against castration and immunocastration” and “Against immunocastration” think that castrating animals is more harmful and worse than consumers classified as “Indifferent”. Moreover, consumers “Indifferent” and “Against immunocastration” considered castration easier than consumers “Against castration and immunocastration”. All these attitudes and beliefs towards castration should have helped to create expectations towards meat from castrated and immunocastrated pigs and can partly explain the differences in expectations obtained by the different clusters of consumers.

No differences between clusters were observed in some of the beliefs and attitudes towards castration studied. This lack of differences can be due to the fact that attitudes and beliefs were not used directly to carry out the segmentation of the consumers and also the impact that tasting the product might have had. Castration has decreased in some countries, mainly due to animal welfare issues [43]. However, in the present work, no differences between clusters were found for the statement “Castration causes pain to the animals” and “Castration is necessary”, and all the scores were close to the intermediate level of the agreement scale tending to agree (between 4.3 and 4.8 and between 4.1 and 4.5, respectively), indicating that they do not have a clear opinion about this statement, or that it is a question that is not seen either as something intrinsically good or intrinsically bad. In addition, the same situation can be observed for the statement “Castration is savage”, where no differences between clusters were found, and all the average scores were around 4 (between 3.9 and 4.4), i.e., “neither agree nor disagree”. Similar results were obtained for these statements by consumers from Easter European countries, such as the Czech Republic, Poland, Slovakia and Slovenia [42]. On the other side, consumers from Bosnia and Herzegovina, Bulgaria, Croatia, North Macedonia, Moldova, Serbia and Ukraine agree with the statement “Castration causes pain to the animals”, while consumers from Bulgaria, Hungary and Romania disagree with the statements “Castration is not necessary” and “Castration is savage” [42]. In another study, on the statement “Castration of male pigs is so painful that I think it should not be done”, consumers from Spain, France, the United Kingdom and Germany tended to agree, while consumers from Italy neither agree nor disagree [24]. Thus, if castration is related to animal welfare aspects, the same practice can be considered positive or negative for the animal welfare by different consumers. When information was provided, consumers associated surgical castration without anesthesia to be a cruel practice, while surgical castration with anesthesia and immunocastration were associated with being free of pain and welfare-friendly [22]. Similarly, no clear opinion about the price and the characteristics related to fatness/leanness of the pork from castrated pigs was observed in all the clusters, with all the average scores being close to the “neither agree nor disagree” score. Similar results were found by consumers for most of the eastern European countries [42]. Moreover, no differences between clusters were obtained in the statement “I think castrating animals is… natural/artificial”. On average, answers tended to be on the “artificial” side, which would agree with a study carried out in Norway, where people that do not accept castration wanted the animals to have a natural life [17]. Furthermore, unnaturalness was also one of the reasons the citizens did not accept immunocastration [17,44].

## 5. Conclusions

In the conditions of the present work, it can be concluded that the sex type affects the acceptability in the blind condition of meat by consumers, with overall liking scores from castrated males’ meat being higher than those from the other sex types evaluated. However, this effect is not the same for all the consumers, and it changes when information about the sex type of the meat is provided before its sensory evaluation. These changes are due to the expectations the consumers have towards the different types of meat together with their sensory experience. The sensory scores and expectations towards meat from different sex types are different depending on the consumers. Some consumers are “Indifferent” towards them. Other consumers are “Against castration and immunocastration” and other consumers are “Against immunocastration”. Thus, the inclusion of the sex type of the pig in the label of the pork at the point of purchase, without providing additional information, might influence the purchasing decision and can be negative for meat from castrated or immunocastrated pigs, especially for the segment of consumers that are against these practices (68.6% of the participants in the present study). Thus, this needs to be considered for marketing purposes to define the most appropriate commercial strategies to minimize this effect.

## Figures and Tables

**Table 1:** Demographic characteristics of the consumers by city and cluster (%).

	Total	Barcelona	Madrid	Cluster 1	Cluster 2	Cluster 3
*n*	252	151	101	79	79	94
City						
Barcelona	59.9			55.7	67.1	57.5
Madrid	40.1			44.3	32.9	42.5
Gender						
Men	46.2	44.0	49.5	49.4	44.9	44.7
Women	53.8	56.0	50.5	50.6	55.1	55.3
Age group						
<36 years old	21.5	22.6	19.8	21.8	19.5	22.8
36–50 years old	36.0	36.3	35.6	32.1	44.2	32.6
>50 years old	42.5	41.1	44.6	46.1	36.4	44.6
Education						
Less than primary	2.0	2.0	2.0	1.3	3.9	1.1
Primary	8.0	9.3	5.9	6.3	9.0	8.5
Secondary	5.6	7.3	3.0	3.8	6.4	6.4
High school or equivalent	47.4	49.3	44.6	51.9	46.2	44.7
University degree	37.1	32.0	44.6	36.7	34.6	39.4
Economic situation ^1^						
Difficult	30.7	28.6	33.6	30.4	32.1	29.8
Neither difficult nor accommodated	32.6	30.7	35.7	31.6	41.0	26.6
Accommodated	36.7	40.7	30.7	38.0	26.9	43.6

^1^ Perceived economic situation was evaluated using a 7-point scale from 1-Difficult to 7-Accommodated. Scores from 1 to 3 were considered as “Difficult”, score 4 as “Neither difficult nor accommodated” and scores from 5 to 7 were considered “Accommodated”.

**Table 2:** Consumer purchasing intention of the loins after tasting them in informed conditions considering all the consumers as a pool and by cluster (assessed in a linear scale from 0:’ Sure that I will not buy it’ to 10: ‘Sure I will buy it’).

	Global	Cluster 1	Cluster 2	Cluster 3
		Indifferent	Against Castration and Immunocastration	Against Immunocastration
CF—Castrated females	5.0 ^ab^	5.0 ^ab^	4.8 ^ab^	5.3 ^b^
EF—Females	5.6 ^a^	4.7 ^ab^	5.6 ^a^	6.3 ^a^
IF—Immunocastrated females	4.4 ^bc^	5.1 ^ab^	4.2 ^b^	4.0 ^c^
CM—Castrated males	5.6 ^a^	5.3 ^a^	5.7 ^a^	5.7 ^ab^
IM—Immunocastrated males	4.2 ^c^	4.1 ^b^	5.0 ^ab^	3.6 ^c^
RMSE	2.7	2.4	2.4	2.2

^a,b,c^ Different letters between sex types within a column indicate significant differences (*p* < 0.05). RMSE: root mean squared error.

**Table 3:** Least-squared means of attitudes and beliefs of consumers toward castration by cluster.

	Overall	Cluster 1	Cluster 2	Cluster 3	RMSE	*p*-Value
		Indifferent	Against Castration and Immunocastration	Against Immunocastration		
Beliefs (1: completely disagree to 7: completely agree)						
Castration causes pain to the animal	4.5	4.6	4.8	4.3	1.8	0.211
Meat of castrated animals is of higher quality	3.8	4.8 ^a^	3.9 ^b^	3.8 ^b^	1.3	<0.001
Meat of castrated animals is more expensive	3.8	4.4	4.1	4.2	1.0	0.266
I am willing to pay a little more for meat from castrated animals	4.5	3.9 ^a^	3.3 ^b^	3.3 ^b^	1.5	0.014
Castration is necessary	4.3	4.1	4.4	4.5	1.5	0.258
Meat of castrated animals is leaner	3.8	4.4	4.1	4.1	0.9	0.028
Castration is savage	4.2	3.9	4.3	4.4	1.6	0.078
I prefer to eat meat of castrated animals	4.3	4.3 ^a^	3.5 ^b^	3.4 ^b^	1.3	<0.001
Chemical castration worsens meat quality	4.7	4.4 ^b^	4.7 ^ab^	5.0 ^a^	1.2	0.002
Castration with vaccines improves meatquality	4.4	4.1 ^a^	3.6 ^b^	3.3 ^b^	1.1	<0.001
Attitudes						
I think castrating animals is… (1: harmful to 7: beneficial)	4.1	4.5 ^a^	3.6 ^b^	3.8 ^b^	1.6	0.001
In my opinion, castration of pigs is… (1: easy to 7: difficult)	3.5	3.1 ^b^	4.0 ^a^	3.4 ^b^	1.4	0.001
Castration of pigs is… (1: bad to 7: good)	4.0	4.5 ^a^	3.7 ^b^	3.8 ^b^	1.3	<0.001
I think castrating animals is… (1: natural to 7: artificial)	4.9	4.6	5.0	5.1	1.7	0.137

^a,b^ Different letters within a row indicate significant (*p* < 0.05) differences between clusters.

## Data Availability

The data presented in this study are available on request from the corresponding author.
